# HSA—Coated Magnetic Nanoparticles for MRI-Guided Photodynamic Cancer Therapy

**DOI:** 10.3390/pharmaceutics10040284

**Published:** 2018-12-17

**Authors:** Petr Ostroverkhov, Alevtina Semkina, Victor Naumenko, Ekaterina Plotnikova, Raisa Yakubovskaya, Stepan Vodopyanov, Artem Abakumov, Alexander Majouga, Michael Grin, Vladimir Chekhonin, Maxim Abakumov

**Affiliations:** 1 Institute of Fine Chemical Technology, Moscow Technological University (MIREA), 86 Vernadsky Avenue, Moscow 119571, Russia; mrp_ost@mail.ru; 2 Department of Medical Nanobiotechnology, Pirogov Russian National Research Medical University, Ostrovitianov str. 1, Moscow 117997, Russia; alevtina.semkina@gmail.com (A.S.); chekhoninnew@yandex.ru (V.C.); 3 Laboratory of Biomedical Nanomaterials, National Research Technological University “MISiS”, Leninskiy Prospekt 4, Moscow 119049, Russia; naumenko.vict@gmail.com (V.N.); stepan.vodopianov@yandex.ru (S.V.); alexander.majouga@gmail.com (A.M.); 4 FSBI NMRRC of the Ministry of Health of the Russian Federation, 2-Y Botkinskiy Proyezd 3, Moscow 125284, Russia; plotnikovaekaterina62@gmail.com (E.P.); raisayakub@yandex.ru (R.Y.); 5 Skolkovo Institute of Science and Technology, Nobelya Ulitsa 3, Moscow 121205, Russia; A.Abakumov@skoltech.ru (A.A.); michael_grin@mail.ru (M.G.); 6 Dmitry Mendeleev University of Chemical Technology of Russia, Miusskaya sq., 9, Moscow 125047, Russia; 7 Department of Chemistry, Moscow State University, Leninskie Gory 1/3, Moscow 119991, Russia

**Keywords:** Photodynamic therapy, iron oxide nanoparticles, human serum albumin, MRI

## Abstract

Background: Photodynamic therapy (PDT) is a promising technique for cancer treatment; however, low tissue permeability for irradiating light and insufficient photosensitizer (PS) accumulation in tumors limit its clinical potential. Nanoparticles are engineered to improve selective drug delivery to tumor sites, but its accumulation is highly variable between tumors and patients. Identifying PS accumulation peak in a personalized manner is crucial for therapeutic outcome. Magnetic nanoparticles (MNPs) provide opportunity for tracking drug accumulation in dynamics using non-invasive magnetic resonance imaging (MRI). The purpose of the study was to evaluate MNP loaded with PS as a theranostic tool for treating cancer in mice xenograft colon cancer models. Methods: MNPs coated with human serum albumin (HSA) were loaded with bacteriochlorine *a*. MRI, atomic emission spectroscopy (AES) and fluorescent imaging were used to study MNP and drug accumulation rates and dynamics in CT26 tumors. Tumor growth curves were evaluated in animals that received PDT at different time points upon MNP systemic injection. Results: Peak MNP accumulation in tumors was detected by MRI 60 min post injection (pi) and the data were verified by AES and fluorescent imaging. Up to 17% of injected dose/g of tissue was delivered to malignant tissues 24 h after injection. Consistent with MRI predicted drug accumulation peak PDT performed 60 min after intravenous injection was more efficient in inhibiting tumor growth than treatment scheduled 30 min and 240 min pi. Conclusions: PS loading on HAS-coated MNPs is a perspective approach to increase drug delivery to tumor site. Tracking for MNP accumulation by MRI can be used to predict drug concentration peak in tumors and to adjust PDT time scheduling for improved antitumor response.

## 1. Introduction

Photodynamic therapy (PDT) is an intensively developed approach for cancer treatment [1]. Having started historically from superficial skin cancer in early 1900s [2], nowadays PDT is used for treating variety of tumors, including lung [3], colorectal [4], gastrointestinal [5], and bladder [6] malignancies. 

PDT relies on three key components: (1) photosensitizer (PS); (2) appropriate wavelength light irradiation; and (3) oxygen in media surrounding tumor cells [7]. Under light irradiation PS molecule absorbs photon and converts from singlet basic energy state (S_0_) to singlet excited state (S_1_). Part of absorbed energy is emitted as fluorescent photon and another part drives PS to excited triplet state (T_1_) [7]. Activated PS molecule can transfer the energy via electrons or directly to O_2_ molecules with conversion of O_2_ molecules to superoxide radical of so-called singlet oxygen [8]. Both mechanisms lead to overproduction of reactive oxygen species (ROS), that damage cell membrane, mitochondria, endoplasmatic reticulum or locally deplete oxygen supply by vascular shutdown [9]. Recently PDT was used to elicit antitumor immunity in combination with check-point inhibitors suggesting that adaptive immune response is also an important part of PDT [10].

Despite some success in treating cancer PDT has its own limitations: (1) low tissue permeability for light; (2) low solubility of most PSs; and (3) insufficient accumulation of the PS in tumor site after intravenous injection [8]. In order to improve PDT efficiency, the following approaches have been suggested: (1) conjugation of PS with a targeting moiety [11]; (2) increasing PS water solubility by chemical modification [12]; (3) using a pro-drug that transforms into an active form in tumor site [13]; and (4) PS delivery via nanoparticles (NPs) [8]. Among listed above the last approach seems to be the most promising for number of reasons. First, many NP are chemically designed to encapsulate poorly water soluble drugs such as PS [14,15]. Second, it is well known that NP can passively or actively target tumors significantly improving drug delivery efficiency and decreasing overall toxic effects [16,17]. For a number of NP-based therapies drug release in tumor site is required; however, in the case of PDT, ROS can be produced even if PS is incorporated inside NP. Finally, NP can be used for monitoring drug biodistribution and accumulation in target tissues via different bioimaging modalities [18]. 

Tracking for drug accumulation in tissues in real time is crucial for scheduling PDT [19]. Due to tumors heterogeneity maximum PS concentration time can differ between tumor types and patients and is also dependent on drug properties. Applying PDT immediately after PS injection when maximum drug concentration in tumor vasculature is expected can be preferential for highly vascularized tumors [19]. In this case, antitumor activity is mainly based on vascular collapse. The major issue with activating PSs in blood flow is non-target ROS production and increased toxicity. PDT performed after drug clearance from systemic blood flow is safer being relied only on tissue accumulated PS that selectively kill cancer cell due to direct phototoxic effect [19]. PDT efficiency would benefit from simple non-invasive diagnostic approach that can help to find PS accumulation peak for each individual tumor. 

Fluorescent imaging can work for monitoring drug delivery to superficial tumors, but the approach is impracticable for majority of cancers. Magnetic nanoparticles (MNP) are known to act as magnetic resonance imaging (MRI) contrast agents, enabling non-invasive detection deep in tissues [20]. Several groups have recently suggested MNP-based theranostic systems for tracking PS in tumors [21,22,23,24,25]; however, none of described therapeutics combines high drug loading capacity with improved delivery to malignant tissues, and prominent MR contrasting in vivo. The aims of the study were: (1) to design a theranostic system based on MNP and bacteriochlorin derivatives that would meet aforementioned requirements for MRI guided photodynamic cancer therapy; and (2) to predict the most effective time for PDT by MNP tracking via MRI.

## 2. Materials and Methods

### 2.1. Materials

Iron acetylacetonate (III) (99.9%), benzyl alcohol (99.8%), *N*-hydroxysuccinimide (NHS), 1-ethyl-3-(3-dimethylaminopropyl) carbodiimide (EDC), human serum albumin (has), phosphate buffer saline (PBS), AND dimethyl sulfoxide (DMSO) were purchased from Sigma-Aldrich, (St. Louis, MO, USA). Monoamine terminated poly(ethylene glycol)hydrochloride (NH2-PEG-OH) was from Creative PEGworks (Chapel Hill, NC, USA). Cellulose centrifugal filter units Amicon® with pore diameter of 30 and 100 kDa (Merck, Kenilworth, NJ, USA) and syringe filters with pore diameter of 0.45 and 0.22 μm (Merck, Kenilworth, NJ, USA) were used to sterilize samples. Desalting of polymers was conducted with mini-column for gel filtration PD-10 (GE HealthCare, Chicago, IL, USA). 

### 2.2. Methods

#### 2.2.1. Synthesis of PEGylated HAS-Coated Magnetic Nanoparticles

Synthesis was carried out according to the procedure described earlier [26]. First, synthesis of MNPs cores by thermal decomposition of iron acetylacetonate (III) in benzyl alcohol was carried. MNP-HSA were prepared as follows: distilled water was added to MNP and pH was adjusted to 11 using 1M NaOH and HSA were added. This mixture was incubated at room temperature and continuous stirring, and then was dialyzed (25 kDa) against distilled water. Then, the HSA coating was crosslinked by the action of glutaraldehyde. MNP-HSA was separated from excess of glutaraldehyde using cellulose centrifuge filters (Amicon, 100 kDa, 2000 rpm, MilliporeSigma (Burlington, MA, USA). Purification of MNP-HSA from the excess of unbound HSA was performed by gel. Coating MNP-HSA by PEG carried out by action of EDC and NHS in PBS. The resulting MNP-HSA-PEG was separated from excess of PEG by gel filtration using column NAP-10 (Sepadex G25, eluent—PBS, GE Healthcare Bio-Sciences, (Pittsburgh, PA, USA).

#### 2.2.2. PS Synthesis

PS samples were prepared as described previously [27]. As a starting compound, it was used natural bacteriochlorophyll *a* which was obtained from the *Rhodobacter capsulatus* bacteria biomass. Then, bacteriochlorophyll *a* acidic hydrolysis yield bacteriopheophorbide. 13^1^-(4-aminobutylcarbamoyl)bacteriochlorin methyl ether (PS molecules) were obtained by action of 1,4-diaminobutane, on bacteriopheophorbide a methyl ether with catalytic amount of *N*,*N*-Diisopropylethylamine (DIPEA) and the mixture was refluxed under positive argon pressure. The reaction was followed spectrophotometrically and by thin-layer chromatography. After 24 h reaction mixture transferred into the separatory funnel, chloroform was added, followed by the addition of distilled water. The organic layer was separated, washed, dried and concentrated. The residue was purified by preparative TLC to yield PS. 

#### 2.2.3. Immobilization of PS on MNP

Immobilization was carried out according previously described method [28]. Briefly, PS was dissolved in DMSO. Then solution of MNP-HSA-PEG in PBS was added to PS dropwise to get DMSO:PBS ratio equal to 1:10. The ratio between amounts of PS and MNP was equal to 1:1. The system was continuously stirred for 12 h. During that time nanoparticles size was measured by DLS. Then reaction mixture was purified from free PS by gel-filtration using column NAP-10 (Sepadex G25, eluent PBS). After purification UV-Vis spectra were performed to measure PS concentration.

#### 2.2.4. MNP@PS Complexes Characterization

UV-Vis spectra were recorded on spectrophotometer Victor X3 (Perkin Elmer, Hopkinton, MAUSA). Dynamic light scattering (DLS) measurements were performed on Zetasizer Nano series (Мalvern, Worcestershire, UK). High resolution transmission electron microscopy (HR-TEM) images were recorded using a Tecnai Osiris microscope equipped with a Super-X detector (Thermo Fisher Scientific, Hillsboro, OR, USA) and operated at 200 kV. For HR-TEM the solutions were diluted by equal amount of distilled water and deposited on a holey carbon-film replica.

#### 2.2.5. Biodistribution of Iron Oxide Core of MNP-HSA@PS by AES

All animal studies were approved by the Ethical Committee of Pirogov Russian State Medical University (protocol ## 25/2017, 26/2017). Eight-to-ten-week-old female BALB/c mice bearing CT26 tumors received MNP-HSA@PS by single tail vein injection at dose of 5 mg iron/kg. Animals were sacrificed at 1, 4, and 24 h after i.v. injection (*n* = 4 for each time point) and tumor, liver, spleen, and kidney were collected and dissolved in concentrated nitric acid (48 h incubation at room temperature). Iron concentration in the tissue samples was measured by inductively coupled plasma-atomic emission spectrometry (Agilent 4200 MP-AES, Santa Clara, CA, USA). Untreated mice (*n* = 4) were used as control to identify endogenous (background) iron concentration. 

#### 2.2.6. MRI

CT26-bearing mice (*n* = 5) were anaesthetized with isofluorane and imaged on ClinScan 7T scanner (Bruker BioSpin, Billerica, MA, USA)before and 1–24 h after 5 mg/kg MNP-HSA@PS i.v. injection. The images were obtained using a 20-cm volumetric coil as a transmitter and a 4-segment surface coil as a receiver of the RF signal. Mice were scanned before and after MNP injection using the following settings: (1) fat-suppressed T2-weighted turbo spin-echo (TSE) images were made in the coronary (TR = 2000 ms, TE= 42 ms, FOV = 35 × 60 mm, base resolution (190 × 320) and transversal planes (TR = 3000 ms, TE= 38 ms, FOV = 21 × 30 mm, base resolution (136 × 192); (2) T2* weighted gradient echo (GRE) images were made in the coronary (TR = 400 ms, TE= 10 ms, FOV = 31 × 40 mm, base resolution (200 × 256) and transversal planes (TR = 400 ms, TE= 10 ms, FOV = 27 × 35 mm, base resolution (200 × 256).

#### 2.2.7. Biodistribution of PS Incapsulated in MNP-HSA @PS

PS biodistribution study was performed ex vivo using fluorescent local spectroscopy (FLS) immediately after euthanizing animals. Fluorescence was registered by a contact method using a laser spectral analyzer for fluorescent diagnostics “LESA-06”. Fluorescence was excited by He-Ne laser (excitation wavelength 632.8 nm, spectral range 400–900 nm). Blood, plasma, skin, liver, kidney, spleen and tumor samples were obtained immediately upon MNP-HSA@PS administration (0 h) and 0.5, 1, 2, 4, 6, and 24 hpi (*n* = 3 for each time point). Untreated mice (*n* = 3) were used as control for measuring tissues background autofluorescence. When the fluorescence was excited in the red region of the spectrum, the integrated fluorescence intensity in the spectral range of 640–900 nm was normalized to the integrated intensity of the backscattered diffuse scattering signal of the exciting laser radiation, thereby determining the normalized fluorescence (FN) of the studied tissues. The accumulation of the PS in tissues was assessed by the maximum FN values at a wavelength corresponding to the maximum fluorescence of PS. For each tissue type mean background fluorescence level (from control group) was subtracted from fluorescence of corresponding tissues measured in MNP-HSA@PS treated animals.

#### 2.2.8. In Vivo Imaging System (IVIS)

CT26 bearing BALB/c mice (*n* = 5) were anaesthetized with isofluorane and imaged using IVIS Spectrum CT (Perkin Elmer, Hopkinton, MA, USA) on 640/680–840 nm and 710/760–840 nm excitation/emission wavelengths before and 0.5–24 h after 5 mg/kg MNP@PS4 i.v. injection.

#### 2.2.9. PDT

PDT was performed on day 7 after CT26 cells subcutaneous injection. Prior to PDT, the tumor volume was in the range of 100–180 mm^3^. Animals were injected with the MNP-HSA@PS (5 mg/kg) and received PDT 30, 60 or 240 min after i.v. injection (*n* = 3 for each group). The LED source was used for remote irradiation (wavelength of 740 ± 28 nm, FGUP SSC NIOPIK, Moscow, Russia) with the energy density 90 J/cm^2^. Before irradiation, animals were anaesthetized with droperidol (FSUE Moscow Endocrine Plant, Moscow, Russia) intraperitoneal injection (0.25 mg per animal). Control animals received the same dose of MNP-HSA@PS without subsequent irradiation. Tumors were measured twice a week and tumor volume was calculated as *V* = ½ × *a*^2^ × *b*, where *a* and *b* were the minimal and maximal tumor caliper measurements. 

## 3. Results

### 3.1. MNP-HSA@PS Characterization

**Figure 1:**
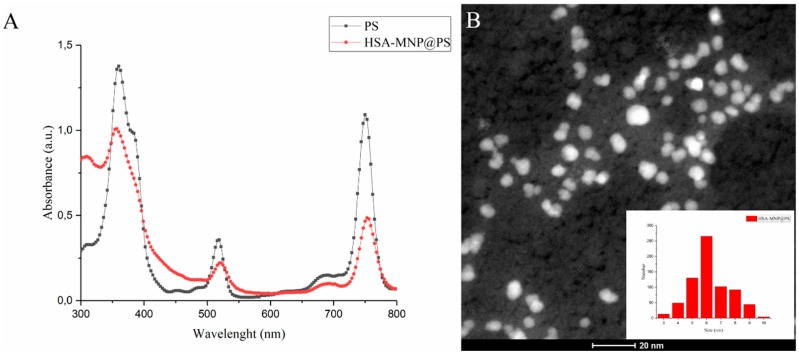
Physical properties of MNP-HSA@PS. Absorption spectrum of PS in DMSO and MNP-HSA@PS in water (**A**); HR-TEM images and size distribution of MNP-HSA@PS (**B**).

First MNP-HSA@PS were characterized by traditional techniques in order to define structure, composition and morphology of obtained nanoparticles. Absorption spectrum (Figure 1A) demonstrated retention of PS photophysical properties in water solution. In addition, loading capacity measured by PS absorption was equal to 22.4 ± 0.9%. To evaluate MNP-HSA@PS size and shape, HR-TEM (Figure 1B) and DLS measurements were performed. Magnetic cores of MNP showed almost monodisperse distribution with average size 6.2 nm (Figure 1B). The hydrodynamic size of MNP-HSA@PS was 83.1 ± 0.6 nm. A detailed characterization of MNP-HSA@PS physical and chemical properties was described previously [28].

### 3.2. Biodistribution of Iron Oxide Core of MNP-HSA@PS

**Figure 2:**
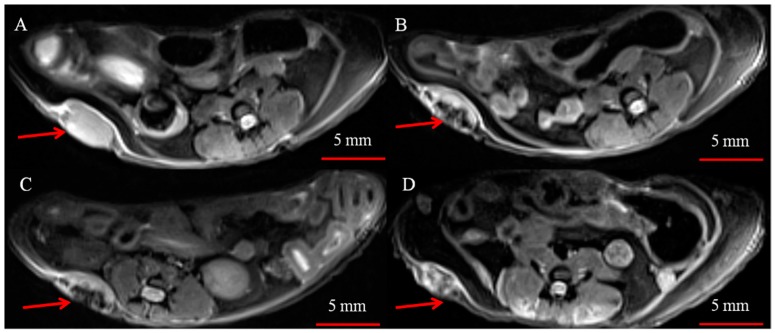
Representative T2 weighted images of subcutaneous CT26 tumors (red arrows) before (**A**) and 1 h (**B**), 4 h (**C**) and 24 h (**D**) after i.v. injection of MNP-HSA@PS.

Next, we studied MNP-HSA@PS accumulation dynamics in tumors using MRI (Figure 2). MNP are known to act as T2 MRI contrast agents shortening T2 relaxation time in target tissues thus leading to decrease in signal intensity. On T2-weighted images tumor region before treatment was clearly seen as hyper-intensive oval shaped structure (Figure 2A). A dramatic increase by 1 hpi and 4 hpi in signal intensity in tumors was observed, suggesting profound MNP-HSA@PS accumulation (Figure 2B,C). After 24 h, tumor tissues were still contrasted but far less than at early time points (Figure 2D).

**Figure 3:**
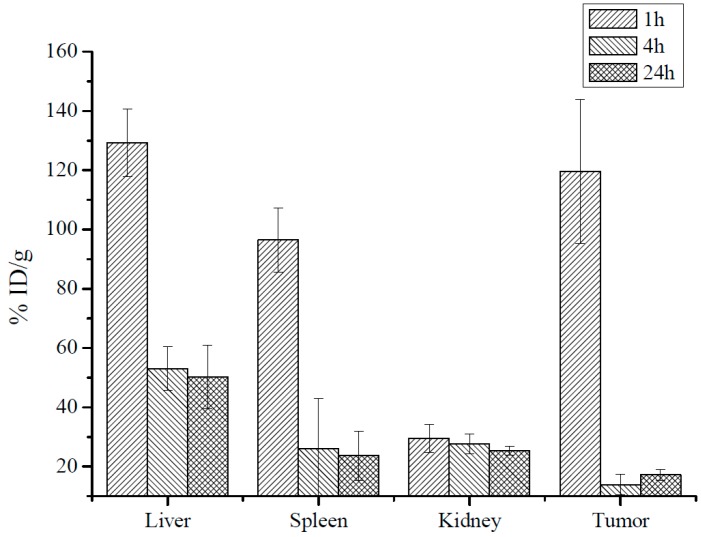
MNP iron oxide core biodistribution after MNP-HSA@PS i.v. injection.

To check if MRI can be used for noninvasive tracking MNP accumulation in tumors we used AES to measure iron concentration in tumors and organs before and 1–24 h post MNP-HSA@PS injection. After subtracting endogenous iron concentration measured in the control (non-treated) group and normalizing to organ weights data was plotted as percentage of injected dose per gram tissue (Figure 3). Maximum iron concentration for all studied samples was detected 1 h after i.v. injection. As expected, liver and spleen accommodated major part of injected dose. Of note, the %ID/g in tumors 1 hpi was comparable to that of liver (129 ± 11 %ID/g and 120 ± 25 %ID/g), pointing to MNP profound accumulation in CT26 tumors. At 4 h iron concentration decreased in liver, spleen and tumors and remained constant up to 24 h after i.v. injection. It should be noted that even at later time points tumors accumulated 14 ± 3 %ID/g (4 h) and 17 ± 2 %ID/g (24 h). Although animals were perfused before collecting organs high iron concentration 1h after i.v. injection was probably due to circulating MNP-HSA@PS. Overall AES results correlated with MRI at 1h and 24 hpi indicating that MRI can be used to predict accumulation peak in tumors. However, being semi-quantitative MRI was not that effective in estimating rapid clearance phase at 4 hpi.

### 3.3. Biodistribution of PS Incapsulated in MNP-HSA@PS

**Figure 4:**
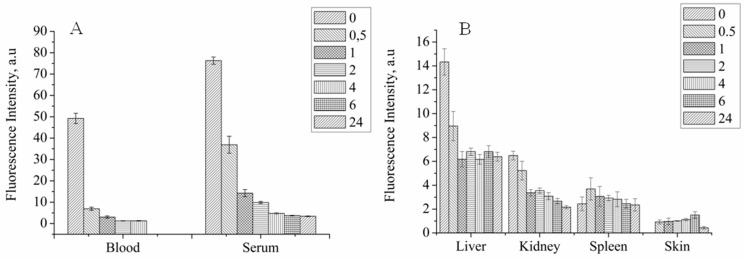
PS biodistribution in systemic circulation (**A**) and organs (**B**) after MNP-HSA@PS i.v. injection.

The concept of non-invasive tracking for PS distribution by MRI is based on assumption that MNP-HSA@PS complexes are stable after systemic injection. If the complex degrades than MNP and PS biodistribution will differ and MRI cannot predict drug accumulation dynamics. To check it we studied PS biodistribution and delivery to tumors by fluorescent imaging (Figure 4A,B).

For blood and serum PS concentration decreased in time dependent manner, showing two-phase curve with first “fast” phase with blood half life time close to 30 min and second “long” phase (Figure 4A). PS concentration in serum was higher than in whole blood for all time points and was still detectable 24 hpi suggesting that MNP-HSA@PS complexes are not bound to cells in systemic circulation. However lower PS signal in whole blood samples can be also due to light scattering from red blood cells that increases background autofluorescence thus limiting method sensitivity. Consistent with AES data, PS was mostly accumulated in liver, spleen and kidneys. Highest PS concentration in organs was detected immediately after MNP-HSA@PS i.v. injection with subsequent decrease and reaching plateau 1–2 hpi. Given that FLS was performed on non-perfused animals the measured fluorescence was coming both from PS circulating in blood flow and PS accumulated in parenchyma. Therefore, high fluorescence detected in organs at early time points (0–30 min) corresponded to high PS concentrations detected in blood flow, while signal detected after 1 h reflected true PS accumulation in tissues. As FLS provides only relative measurement of fluorescence intensity, it can be used for tracking PS kinetics, but not for calculating % of ID.

**Figure 5:**
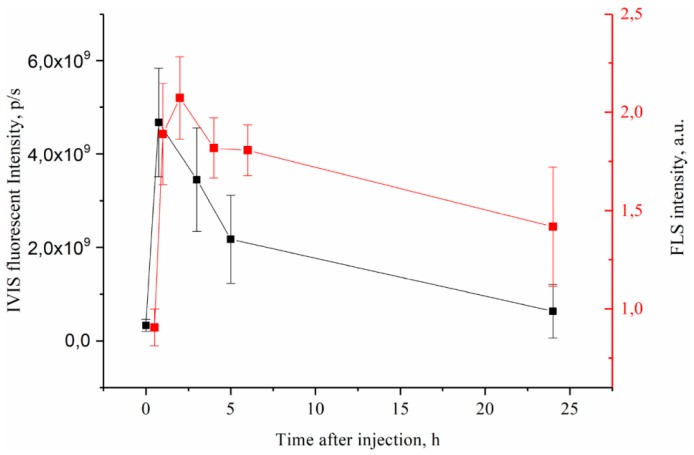
PS accumlation in CT26 tumors after i.v. MNP-HSA@PS injection.

PS accumulation in tumor was measured by two independent techniques: FLS and IVIS (Figure 5). Both methods demonstrated similar trend in fluorescence intensity during monitored period with accumulation peak at 1–2 h after injection and then gradual decrease to background levels at 24 hpi. Most importantly, the PS delivery profile in tumors was consistent with MNP accumulation dynamics (see Figure 4 and Figure 5), suggesting that MNP and PS behaved like a single complex in vivo and were delivered together to tumor site. This result is very important for validating MRI as diagnostic technique for predicting drug accumulation peak in tumors that in turn is crucial for scheduling PDT.

### 3.4. PDT Therapy in Mice

**Figure 6:**
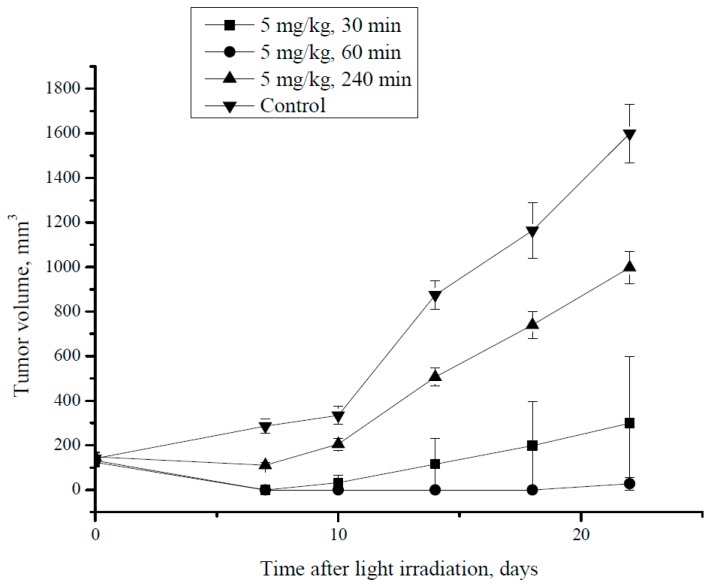
CT26 tumor growth dynamics after PDT at different time intervals i.v. post MNP-HSA@PS injection.

Finally, we wanted to prove that synthesized MNP-HSA@PS can be used as a theranostic tool to identify by MRI drug accumulation peak in malignant tissues for applying PDT. Based on MRI data we selected three time points with optimal (1 hpi) and suboptimal (30 min, 240 min) drug accumulation in CT26 tumors. Mice that received MNP-HSA@PS without irradiation served as control (Figure 6).

During the first week after treatment, all animals that received PDT demonstrated delayed tumor growth comparing to control group. Thus, at day 7 tumors were not found in mice irradiated 30 min and 60 min after MNP-HSA@PS injection, whereas in 240 min group and control the tumor size was 110.6 ± 11.3 mm^3^ and 286 ± 32 mm^3^, respectively (*p* < 0.05). However, starting from day 10 tumor regrowth was observed in animals that received PDT 240 min after i.v. injection. Both groups that were irradiated shortly after drug administration (30 min and 60 min) demonstrated profound tumor growth inhibition during monitored period compared to controls. Although there was no significant differences in tumor growth kinetics between 30 min and 60 min group, for the former one relapse was detected at day 14, while in the latter all animals were free of tumors up to day 22. The 240 min group showed significantly higher tumor volume (998.2 ± 72.1 mm^3^) in comparison to the 30 min and 60 min groups (*p* < 0.05). However, tumor volume in 240 min group was still lower than in controls (1598 ± 133 mm^3^; *p* < 0.05). Overall the results provide proof of principle for using MNP-HSA@PS as a theranostic tool for improved PDT.

## 4. Discussion

Theranostics is a relatively new concept in PDT therapy. Despite the approach has shown promises in number of studies some key requirements are still unmet. For instance, drug loading capacity [25] as well as drug delivery to tumors [23] needs to be optimized for improved PDT. Also, high MR contrasting is important to identify precisely drug accumulation peak in tumors. We have previously described theranostic system for PDT, combining bacteriochlorine *a* and iron oxide nanoparticle coated with HSA [28,29]. Based on in vitro properties it has been hypothesized that the MNP-HSA@PS can be used to identify therapeutic window for PDT and to inhibit tumor growth.

Loading capacity identified as ratio between loaded drug and overall mass of the system was much higher (22.4 ± 0.9%) than previously described for similar delivery system (1.54%) [25]. Due to high hydrophobicity PS in aqueous media [30] tend to form aggregates with impaired photoactivity. However comparison between MNP-HSA@PS and molecular PS absorbance spectra did not reveal any changes suggesting that PS retained its photoactivity in the complexes. Based on iron oxide core size (6.2 nm) and hydrodynamic diameter of MNP-HSA@PS (83.1 nm) we hypothesized that each complex consisted of several MNPs encapsulated in HSA coating with PS molecules absorbed in HSA shell.

To understand behavior of both vehicle and drug MNP-HSA@PS biodistribution and delivery to tumors were studied in dynamics. After systemic administration MRI identified MNP accumulation peak in tumors around 1 hpi that was confirmed by AES. Moreover this time point coincided with PS maximum accumulation in tumors supporting the idea that MRI can be used for following PS in vivo. Profound tumors T2 contrasting suggested MNP-HSA@PS high delivery efficiency and indeed iron concentration measurements revealed that 1 h after administration tumors accumulated up to 7.8% of total injected dose, that was much higher than previously reported for most of NP [31].

Having all three components improved (loading capacity, MRI contrasting and % delivery) we sought to check how the theranostic tool worked for PDT. Consistent with MRI data tumors irradiation at early time points (0.5–1 h) was more efficient in inhibiting tumor growth than PDT performed 6 hpi. Total amount of drug in tumor includes both intravascular (circulating) and extravasated (interstitial) fractions and PS activation in these two compartments is responsive for vascular shutdown and direct cytotoxic effect, respectively. Typically MNP maximum concentration in blood is observed immediately upon injection followed by gradual decrease with circulation half-lives depending on MNP physical characteristics and surface modification. Thus, a 10-fold drop in MNP-HSA@PS blood concentration was found 1 h after treatment. Based on PS pharmacokinetics in blood one can suggest that applying PDT immediately after MNP administration is a universal approach. Nevertheless, irradiation 1h after injection tended to provide better response comparing to 30 min group. More important irradiating PS in blood flow may result in cytotoxic effects downstream the tumor. In preliminary experiments PDT scheduled 30 min after MNP-HSA@PS administration with superior energy density (150 J/cm^2^) led to severe toxicity while no side-effects were observed when irradiation was performed at later time points (data not shown). In addition, it should be noted that most of xenograft tumors (including CT26) are vascularized only on the edges and have vast hypoxic core areas. In this case (opposite to highly vascularized tumors) vascular collapse is not a primary mechanism of antitumor response and most likely PS accumulation in tissue determines PDT outcome. MNP passive delivery to tumor is based on enhanced permeability and retention effect and MNP concentration in malignant tissues increases in time dependent manner before reaching plateau. As PS tissue accumulation increases with time while PS blood level drops down a trade-off should be found to ensure both safety and antitumor response. The optimal time interval between nanodrug administration and irradiation may differ between tumors, patients, and even in each individual tumor with time, so a personalized approach is highly required to improve PDT efficiency. 

## 5. Conclusions

In current work we have shown that HSA-coated MNPs: (1) efficiently immobilize PS molecules; (2) deliver up to 17% of injected dose/g to tumors 24 hpi; (3) enable precise MRI tracking for drug accumulation in malignancies; and (4) can be used to adjust irradiation time for the most prominent tumor growth inhibition. Overall, the results suggest that synthetized MNP-HSA@PS combined with MRI imaging is a perspective approach for PDT based cancer theranostics.
